# An Improved DQN Framework with Dual Residual Horizontal Feature Pyramid for Autonomous Fault Diagnosis in Strong-Noise Scenarios

**DOI:** 10.3390/s25247639

**Published:** 2025-12-16

**Authors:** Sha Li, Tong Wang, Xin Xu, Weiting Gan, Kun Chen, Xinyan Fan, Xueming Xu

**Affiliations:** 1 School of Mechanical Engineering, Shanghai Dianji University, Shanghai 201306, China; lisha@sdju.edu.cn (S.L.); fanxy@sdju.edu.cn (X.F.); xuxueming0217@163.com (X.X.); 2 State Key Laboratory for Manufacturing Systems Engineering, Xi’an Jiaotong University, Xi’an 710049, China; ganweiting@stu.xjtu.edu.cn (W.G.); chenkun@mail.xjtu.edu.cn (K.C.); 3 School of Mechanical Engineering, North University of China, Taiyuan 030051, China; ninaxx79@163.com

**Keywords:** strong-noise data, deep reinforcement learning, fault diagnosis, feature pyramid network

## Abstract

Fault diagnosis methods based on deep learning have made certain progress in recent years. However, in actual industrial scenarios, there are severe strong background noise and limited computing resources, which poses challenges to the practical application of fault diagnosis models. In response to the above issues, this paper proposes a novel noise-resistant and lightweight fault diagnosis framework with nonlinear timestep degenerative greedy strategy (NTDGS) and dual residual horizontal feature pyramid (DRHFPN) for fault diagnosis in strong noise scenarios. This method takes advantage of the strong fitting ability of deep learning methods to model the agent in reinforcement learning by the ways of parameterization, fully leveraging the advantages of both deep learning and reinforcement learning methods. NTDGS is further developed to adaptively adjust the action sampling strategy of the agent at different training stages, improving the convergence speed of the network. To enhance the noise resistance of the network, DRHFPN is constructed, which can filter out interference noise at the feature map level by fusing local feature details and global semantic information. Furthermore, the feature map weighting attention mechanism (FMWAM) is designed to enhance the weak feature extraction ability of the network through adaptive weighting of the feature maps. Finally, the performance of the proposed method is evaluated in different datasets and strong noise environments. Experiments show that in various fault diagnosis scenarios, the proposed method has better noise resistance, higher fault diagnosis accuracy, and fewer parameters compared to other methods.

## 1. Introduction

Rotating machinery plays a crucial role in modern industry and is widely used in multiple fields, such as aerospace and new energy [1]. However, its operating conditions are harsh and complex, which makes it extremely prone to malfunction [2]. To ensure the operational stability and reliability of mechanical equipment and reduce safety issues and economic losses caused by equipment failures, it is of great significance to carry out research on fault diagnosis of rotating machinery [3,4].

To achieve fault diagnosis of mechanical equipment accurately, many scholars have proposed fault diagnosis methods based on signal processing [5,6,7], such as adaptive mode decomposition [8], complete ensemble empirical mode decomposition with adaptive noise (CEEMDAN) [9], and wavelet packet decomposition [10]. These methods are commonly employed to process original signals by extracting the intrinsic mode functions (IMFs) from the original signals [11] and filtering out noise by using metrics like kurtosis [12], the Gini coefficient [13], and the correlation coefficient [14]. Subsequently, fault diagnosis is achieved based on methods like fast Fourier transform (FFT) [15], envelope spectrum analysis [16], and so on [17,18,19,20]. The above methods have made certain contributions to the field of fault diagnosis. However, mechanical equipment has numerous operating parameters, complex fault mechanisms, and a huge amount of status data. These methods require high levels of expert experience and have difficulty meeting the end-to-end fault diagnosis needs of actual industrial environments.

With the development of deep learning technology, fault diagnosis methods based on neural networks are proposed to meet the end-to-end fault diagnosis requirements of actual industrial scenarios [21,22,23]. Wang et al. [24] employed a spatial mapping method to extract fault feature information from noisy data, combining it with the Wasserstein distance to continuously optimize extracted features, achieving fault diagnosis well on strong noise data. However, the limited computing speed of this method restricts its application in real-time fault diagnosis scenarios. Zhang et al. [25] constructed a feature enhancement module based on an attention method to enhance network noise resistance, and finally achieving fault diagnosis in strong-noise data by combining residual network (ResNet) and selective kernel convolution, but the method has a certain degree of hyperparameter problem. Chen et al. [26] combined residual connections and an attention method in channel as well as spatial scales to achieve a bearing fault diagnosis with strong-noise data. The above methods have improved the accuracy of fault diagnosis to a certain extent. However, they mainly rely on the huge number of parameters of deep learning models to forcibly fit the data distribution, which is not practical enough in actual industrial environments with limited computing resources.

In response to the above problems, many scholars have proposed fault diagnosis methods based on deep reinforcement learning (DRL). Li et al. [27] designed a multi-objective network to enhance the stability of the agent model during the policy learning process and utilized a generative model to achieve a fault diagnosis of rotating machinery in small sample scenarios. Xia et al. [28] achieved fault diagnosis of a motor by combining deep reinforcement learning and a mirror prototypical network. To improve stability during the agent training process, Li et al. [29] enhanced a dense neural network using multi-scale hybrid attention and implemented bearing fault diagnosis with an actor-critic. Zhao et al. [30] proposed a two-stage fault diagnosis framework, in which comparative loss is utilized to reduce intra-class distances and increase inter-class distances during the pre-training phase and adaptive reward is used during the fine-tuning phase, significantly improving the model generalization and robustness in the fault diagnosis process. Although the above-mentioned fault diagnosis methods based on reinforcement learning have shown certain effects, the strong noise interference in actual industrial scenarios is severe, which makes it difficult to extract fault features and also interferes with the strategy optimization process of the agent, limiting the application of these methods in the actual environment.

In response to the above issues, this paper proposes a novel noise-resistant and lightweight fault diagnosis framework with a nonlinear timestep degenerative greedy strategy (NTDGS) and a dual residual horizontal feature pyramid network (DRHFPN) for fault diagnosis in strong noise scenarios. This method utilizes the strong fitting ability of deep learning to parametrically model the action of the agent model in reinforcement learning, thereby fully leveraging the advantages of both deep learning and reinforcement learning models, and NTDGS is proposed to optimize the action sampling strategy of the agent at different training stages. Furthermore, a DRHFPN and feature map weighting attention mechanism (FMWAM) are proposed to enhance the noise resistance and weak feature extraction ability of the network. The specific contributions of this paper are as follows:

(1) NTDGS is proposed to adaptively adjust the action sampling strategy of the agent model at each training stage, achieving a balance between the agent’s exploration and utilization of the external environment and improving the network convergence speed.

(2) To enhance the noise resistance and robustness of the network, a DRHFPN that can filter out interference noise at the feature map level by fusing local feature details and global semantic information is developed.

(3) An innovative attention mechanism FMWAM that can enhance the weak feature extraction ability of the network by weighting different feature maps during the convolution process is constructed.

(4) A novel noise-resistant and lightweight fault diagnosis framework that combines the advantages of deep learning and reinforcement learning models and has better noise resistance, higher fault diagnosis accuracy, and fewer parameters is proposed.

The structure and organization of this paper are as follows: Section 2 introduces the basic theories involved in the method proposed in this paper. Section 3 provides a detailed explanation of the proposed method. Section 4 describes the experimental data and evaluates the performance of the proposed method in different datasets and strong noise scenarios. The main conclusion of this paper is in Section 5.

## 2. Deep Q-Network

As a milestone algorithm in the field of deep reinforcement learning (DRL), the deep Q-network (DQN), which combined with deep learning algorithms utilizes the data generated by the interactions between agent and the environment and the powerful data fitting ability of neural networks to estimate the Q-value, achieves an end-to-end network architecture from perception to decision-making.

DQN is an enhancement of the Q-learning algorithm that is a classic temporal difference (TD) algorithm in the field of RL Traditional Q-learning. It is primarily used for scenarios in which both the state and action spaces are discrete and finite. It is worth pointing out that Q-learning is considered an off-policy algorithm because $a^{\prime }$ is not determined online by the greedy policy in state s′. Its optimized expression is as follows:

(1) $$Q\left(s,a\right)\leftarrow Q\left(s,a\right)+\alpha \left[r+\gamma \underset{a^{\prime }\in A}{\max}Q\left(s^{\prime },a^{\prime }\right)-Q\left(s,a\right)\right]$$

where s is the initial state, a is the action selected according to the current policy in state s, and r represents the feedback reward by taking action a in state s. $s^{\prime }$ denotes the new agent state after executing action a in state s. γ denotes a reward discount factor; the larger it is, the more the agent pays attention to long-term cumulative rewards. A smaller value indicates that the agent is more focused on immediate rewards. Q(s,a) is the action-value function, the learning rate is expressed as α, and $\underset{a^{\prime }\in A}{\max}Q\left(s_{i}^{\prime },a^{\prime }\right)$ is the maximum action-value among all the action-value in $s_{t+1}$.

As a main algorithm in the field of DRL, DQN can achieve a good estimation of Q-value based on the powerful function-approximation capabilities of the neural network and the data generated by the interaction process between agent and environment. Specifically, when both the state and action are continuous, the neural network takes the state s and action a as network input and the estimated value obtained by taking action a in state s as output. When the action space is discrete, the network can either follow the approach used for continuous action space or use only the state s as input. In this case, the output is the Q-value estimated by the neural network for each possible action in the current state. The loss function of the Q-network in DQN is expressed as follows:

(2) $$\omega *=\arg\underset{\omega }{\min}\frac{1}{2N}{\left[Q_{\omega }\left(s_{i},a_{i}\right)-\left(r_{i}+\gamma \underset{a^{\prime }\in A}{\max}Q_{\omega }\left(s_{i}^{\prime },a^{\prime }\right)\right)\right]}^{2}$$

where ω represents the neural network parameters, N represents the number of samples, and $\;\arg\underset{\omega }{\min}\left(\cdot \right)\;$ denotes the value of ω that minimizes the function.

In general, the training data for the neural network needs to be independent identically distributed that is, the training data should follow the same distribution and be independent of each other. However, in Q-learning, the data collected through the Markov decision process (MDP) does not satisfy this independence assumption. It means that the current state is dependent on the previous state, which can cause the network to overfit to the data sampled recently during training. In addition, the sample utilization efficiency of Q-learning is not high, and each data sample will only be used once. DQN is an improvement of Q-learning, which proposes an experience replay strategy to store and sample training data, breaking the correlation between samples, meeting the independence assumption required for network training, and effectively improving sample utilization efficiency. Specifically, the experience replay strategy refers to storing tuples of data that involves the state, action, reward, and next state obtained by the interaction between agent and environment into the replay buffer. When the number of samples in the replay buffer meets a certain condition, the data is randomly sampled from the buffer and input into the Q-network for training.

Furthermore, the network output is included in the loss function of Q-learning, which makes the training target of the network change simultaneously, leading to training instability. To address this issue, DQN employs a dual-network structure consisting of a Q-network and a target network for updating the training parameters. The specific parameter updating strategy is as follows.

1. The Q-network $Q_{\omega }(s,a)$ is used to compute $Q_{\omega }\left(s,a\right)$ in Equation (2). The training parameters of the Q-network are then updated and optimized by the backpropagation algorithm.
2. The target network $Q_{\omega^{-}}(s,a)$ is employed to calculate $r+\gamma \underset{a^{\prime }\in A}{\max}Q_{\omega^{-}}\left(s^{\prime },a^{\prime }\right)$ in Equation (2). The parameters of the target network are synchronized with the Q-network every N steps to ensure training stability.

## 3. The Proposed Fault Diagnosis Method

### 3.1. Nonlinear Timestep Degenerative Greedy Strategy

Greedy strategy is an action sampling method in reinforcement learning. The fully greedy strategy is to take the action with the highest expected reward at each state, which is purely utilization and can cause the model to converge to a local optimum in the early stages of training. The ϵ-greedy method employs a constant that considers the future potential value of other actions and allows the agent to take other actions with a certain probability. However, as the exploration continues, the agent estimates the rewards that will be obtained by taking each action more and more accurately. At this point, it is necessary to consider reducing exploration and focusing on utilization. Therefore, a more adaptive action sampling method is required. In response to the above problems, NTDGS is proposed in this paper.

(3) $$a=\{\begin{array}{ll} randint(A), & P=\epsilon \\ \arg\underset{a\in A}{\max}Q\left(s,a\right), & P=1-\epsilon \end{array}$$

where A is the set of all possible actions, randint(A) denotes the random selection of an action from A, P is the probability of selecting the action, ϵ is a constant, which indicates the probability of selecting an action randomly, and the $\arg\underset{a\in A}{\max}Q\left(s,a\right)$ is the value of action a when the action-value function  Q(s,a) is maximized.

Specifically, NTDGS can adaptively adjust the action sampling strategy at different training stages of the agent and follow a deterministic strategy for action sampling when the model approaches convergence, fully ensuring the balance between the agent’s exploration and utilization of environmental knowledge at different training stages. The specific expression of this strategy is as follows:

(4) $$a=\{\begin{array}{ll} randint(A), & P=\frac{1}{n^{2}+1}\\ \arg\underset{a\in A}{\max}Q\left(s,a\right), & P=1-\frac{1}{n^{2}+1} \end{array}$$

where n is an adaptive degradation factor, which can adjust the sampling strategy of the action to adapt to different training stages.

From the above formula, it can be seen that with continuous training, the exploration process is basically completed. Then, through the degradation factor n, it adaptively adjusts the action sampling strategy and degenerates into a completely greedy strategy when the model converges, which ensures that the agent can fully utilize the effective information obtained through exploring the environment in the early stage of training.

### 3.2. Dual Residual Horizontal Feature Pyramid Network

**Figure 1:**
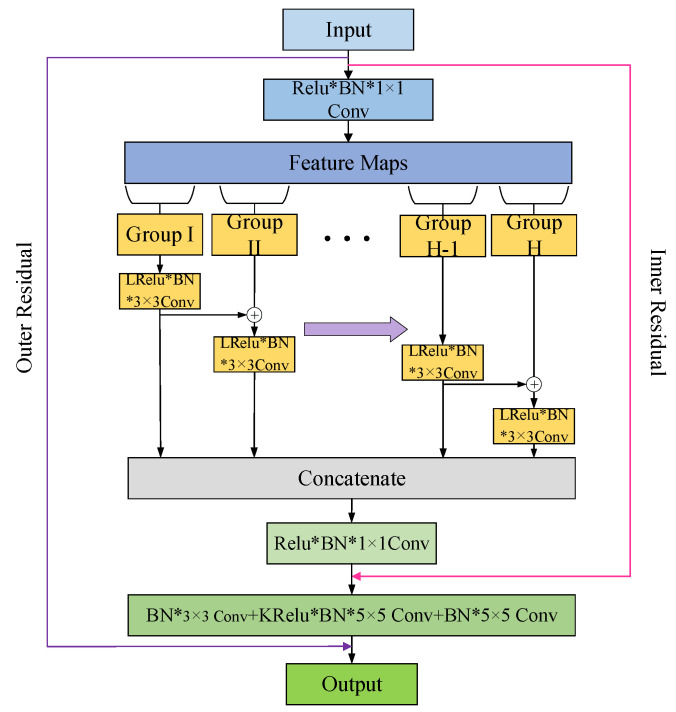
The specific structure of dual residual horizontal feature pyramid network.

In this section, DRHFPN is developed to extract local details and global semantic information for the isomorphic fusion of multi-fine-grained features, thereby filtering out the strong background noise hidden in the original vibration signals and enhance the noise resistance and robustness of the network. Specifically, this module is designed as a horizontally stacked structure to progressively extract the delicate feature information contained in the data, gradually forming a feature pyramid network structure. Then, multi-fine-grained features from different channel groups are fused to enable the network to synthesize multi-scale feature information, which enlarges the receptive field and enhances the network’s ability to extract global abstract fault features and filter noise interference from original data. The specific structure of DRHFPN is shown in Figure 1.

As can be seen in Figure 1, original feature data is initially processed using a 1 × 1 convolution kernel, followed by BatchNorm and Relu, before being sent to the subsequent network for feature map grouping. The feature map is divided into H groups. After passing through Group I, the receptive field of the feature data in each group gradually increases, forming a feature pyramid structure that effectively expands the receptive field. The principle of this process is as follows:

(5) $$\vartheta_{i}=\sum_{i=2}^{H}F_{i}\left(F_{i-1}\left(\zeta_{i-1}\right)+\zeta_{i}\right)$$

where $\zeta_{i}$ is the feature maps of the i-th group, $F_{i}\;$ is the nonlinear mapping function of the i-th group of feature maps, $\vartheta_{i}$ denotes the final output of the i-th group of feature maps, and H represents the number of feature map groups. The calculation formula can be expressed as follows:

(6) $$H=\frac{R}{C}$$

where R is the total number of feature maps during the convolution process, C denotes the number of feature maps in each group, and H symbolizes the number of feature map groups.

In addition, to alleviate the vanishing gradient problem that occurs in the network training process, residual connections are employed to merge the original input features with the multi-fine-grained features processed by the feature pyramid network. At the same time, considering that a single residual connection cannot effectively achieve global identity mapping, this network is combined with two asynchronous residual branches to reuse the feature data. Specifically, the inner residual is deployed to merge the original input features with the multi-fine-grained features processed by the feature pyramid network, and the outer residual is used to fuse the aggregated features processed by the inner residual, achieving asynchronous composite identity mapping of multi-fine-grained features and effectively preserving the details and semantic information in the original data.

### 3.3. Feature Map Weighting Attention Mechanism

During network training, strong background noise in the original signals can overwhelm weak fault features. In order to further enhance the noise resistance and robustness of the network, a feature map weighting attention mechanism (FMWAM) is proposed to improve the weak feature extraction ability of the network. Analogous to the way the human brain thinks when analyzing things, the main principle of this method is to increase the weight of important information during the training process while reducing the weight of noisy information.

Specifically, this method first extracts the dominant features on each feature map to construct a weight vector, then processes the vector using a normalization function to obtain the weighting coefficients of each feature map. Finally, based on this weight coefficients, attention weighting is applied to each feature map. This method can make the network pay more attention to dominant features during the convolution process and reduce the influence of interference noise, thereby enhancing the network’s noise resistance and robustness. The calculation formula for this method is as follows:

(7) $$Y=\sigma \left(\varphi_{max}^{i}(x)\right)\varphi_{i}(x)\;\;\;i\in \left[0,G-1\right]$$

where x is the input, $\varphi_{i}(x)$ denotes the *i*-th feature map in the process of convolution operation, $\varphi_{max}^{i}(x)\;$ is the maximum value in the *i*-th feature map, and G represents the number of feature maps. σ(·) is used for normalizing the weight vector, and it can be expressed as follows:

(8) $$softmax\left(x\right)=\frac{e^{\varphi_{max}^{i}(x)}}{\sum_{j=0}^{j=G-1}e^{\varphi_{max}^{j}(x)}}$$

### 3.4. Fault Diagnosis Process for Strong-Noise Data

**Figure 2:**
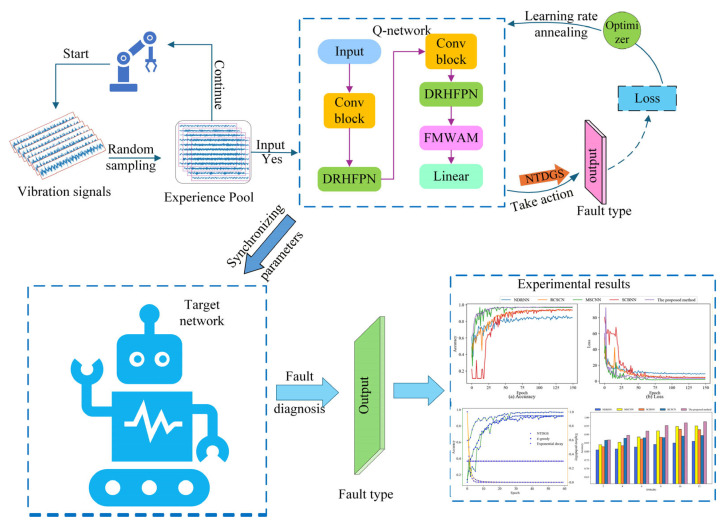
Fault diagnosis process based on the proposed method.

To achieve accurate fault diagnosis in strong noise scenarios, this paper proposes a novel noise-resistant and lightweight fault diagnosis framework with NTDGS and DRHFPN for fault diagnosis in strong-noise scenarios. The above sections introduce the main components of the proposed method, and this section provides a detailed explanation of the fault diagnosis process based on this proposed method, as shown in Figure 2.

To address the challenge of fault diagnosis under strong-noise data, IDQN was developed to achieve autonomous decision-making fault diagnosis with DRHFPN in this paper. In this method, the strong background noise in the raw signal data is filtered out and high-level abstract features are extracted to enable fault diagnosis in scenarios with strong background noise. The pseudo-code of the proposed method is shown in Algorithm 1.
**Algorithm 1:** Pseudo-Code of the Proposed Method
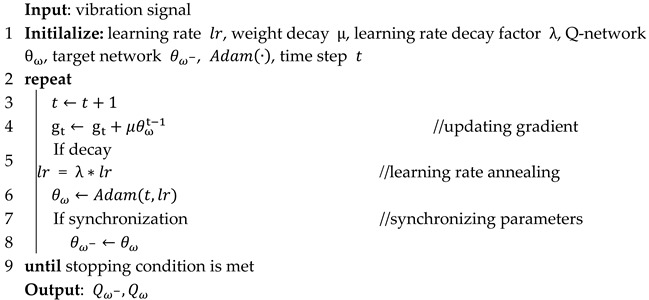


For the detailed fault diagnosis process, the raw data is first divided into two parts for training and testing. The training data is sampled into the experience replay pool using the timestep degenerative greedy strategy proposed in this paper. When the number of samples in the pool meets the sampling conditions, the Q-network is trained, and the target network parameters are updated and synchronized at regular intervals. The detailed fault diagnosis process can be seen from Figure 2.

It is worth noting that this paper redefines terms used in reinforcement learning models such as reward, action, and state to match fault diagnosis tasks. Specifically, state s represents the vibration signal and action a is the specific fault type, which can be expressed as a single heat vector [0,1,2,…,M−1], where M is the number of fault types. Reward is defined as feedback on the fault diagnosis result, with 1 for accuracy and −1 for error. During the network training process, the training data will be randomly shuffled, and the number of samples of each type will be evenly distributed. Therefore, after performing action a in the current state s, the transition probability to the next state $s^{\prime }$ is $\frac{1}{M}$, which has no correlation with the state at the previous moment. In this case, this process still possesses the Markov property.

In addition, in order to enhance stability during the network training process, a dynamic learning rate annealing strategy is employed to optimize the network training process. This strategy can adaptively decrease the learning rate when the output loss is near the global minimum, thereby accelerating network convergence. In this method, the initial learning rate is attenuated at predetermined intervals, such as after a fixed number of epochs. The learning rate decays at a set proportion until the network converges. This approach ensures that network parameters are quickly updated with a large learning rate during the early stages of training and that the global optimal solution is found with a low learning rate as the network approaches convergence, thereby effectively improving network convergence speed. The detailed formula of the method is as follows:

(9) $$lr=\{\begin{array}{ll} \lambda lr_{i}, & i=E,2E,3E,\ldots \\ lr_{i}, & otherwise \end{array}$$

where $lr_{i}$ denotes the learning rate of the network at the i th epoch, λ is the learning rate decay factor, and E is the number of intervals at which the learning rate decays, measured in epochs.

Finally, an output normalization strategy is employed to optimize the overall training objective during backpropagation. This strategy ensures that the network maximizes the logits when predicting target data, improving classification accuracy. By maximizing the difference in feature distribution between the target and other classes, this approach enhances the stability and robustness of network training. The total loss formula is expressed as follows:

(10) $$L=\frac{MSE\left(Q_{\omega }^{norm}\left(s_{i},a_{i}\right),r_{i}+\gamma \underset{a^{\prime }\in A}{\max}\left(Q_{\omega^{-}}^{norm}(s_{i}^{\prime },a^{\prime }\right)\right)}{N_{sample}}$$

where ω and $\omega^{-}$ donate the model parameters of the Q-network and the target network, respectively. MSE(·) denotes the mean square error loss function, $Q_{\omega }^{norm}$ indicates that the output of the Q-network is normalized using softmax(·), and $N_{sample}$ represents the number of samples.

## 4. Experiments and Result Analysis

### 4.1. Experimental Data Overview

**Figure 3:**
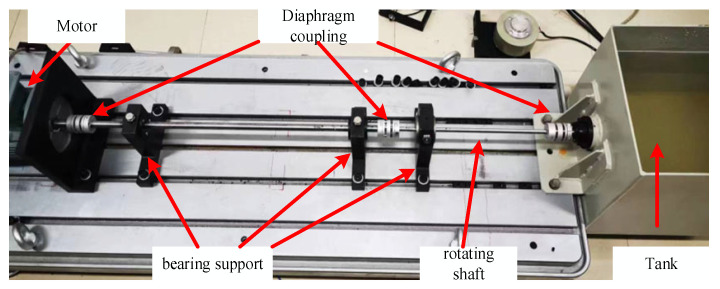
Fault diagnosis experimental platform.

To validate the effectiveness of the proposed method, a fault diagnosis platform for ship shafting was constructed. This platform comprises a motor, transmission shaft, diaphragm coupling, sliding bearing, and bearing housing. It is also equipped with a water tank and a propeller to simulate the actual operating conditions of a ship, as shown in Figure 3. In this fault diagnosis platform, the length of the transmission shaft is 1100 mm. The long and short shafts measure 770 mm and 330 mm in length, with diameters of 15 mm and 10 mm, respectively. The long shaft is connected to the motor, while the short shaft is attached to the propeller in the water tank. Both shafts are supported by a bearing housing, and the coupling used is a diaphragm coupling. The stern shaft has a length and diameter of 100 mm and 10 mm, respectively.

**Figure 4:**
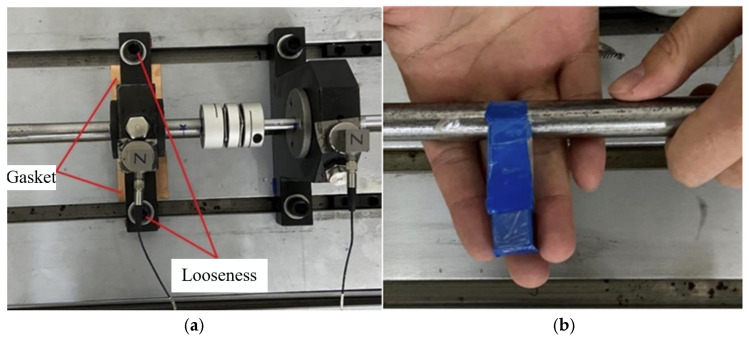
Faults in the ship shafting system. (**a**) Looseness fault and misalignment fault. (**b**) Unbalance Fault.

In this platform, the middle bearing and the bearing on the thin wall of the water tank were selected as the mounting positions for the two three-way vibration accelerometers. The rotational speed of the bearing is 1200 rpm, with a sampling frequency of 5000 Hz in the experiment. To minimize the impact of interference errors on the experimental process, the fixing torque of the anchor bolts was adjusted to 30 N·m for each experiment. The primary fault conditions simulated in this experiment include shaft misalignment, loose intermediate bearing seat, and imbalance. Misalignment faults were simulated by placing copper sheets of varying thickness at both ends of the intermediate plain bearing grounding. The loose fault condition was simulated by adjusting the torque of the fixing bolt on the bearing seat using a torque wrench, with the loosened torque set to 10 N·m. Imbalance faults are simulated by attaching a mass to the coupling. The schematic diagram of each type of fault is shown in Figure 4.

**Figure 5:**
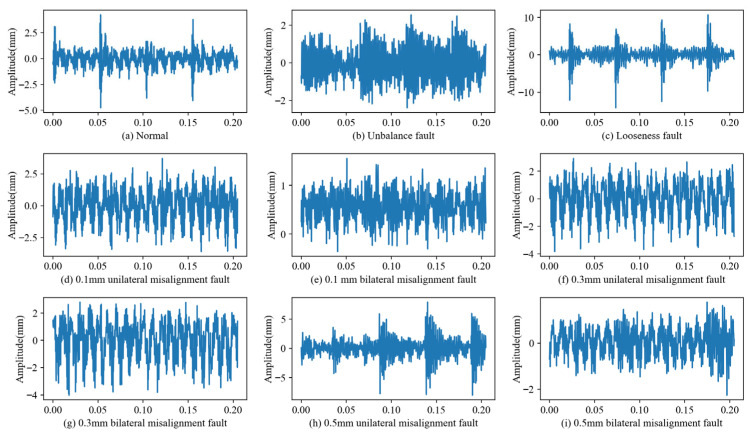
Time-domain vibration signals of ship shafting in different states.

To fully verify the effectiveness of the proposed method, nine different fault types were selected in this section. These included the looseness fault of the bearing seat, the imbalance fault, unilateral misalignment faults of the shafting system at 0.1 mm, 0.3 mm, and 0.5 mm, bilateral misalignment faults at 0.1 mm, 0.3 mm, and 0.5 mm, and the normal state of the shafting system. In following experiments, each sample consists of 1024 data points, and each fault type has 320 samples, resulting in a total of 2880 samples. The ratio of training set to test set is seven to three, the detailed information about the experimental data can be seen in Table 1, and the time-domain waveforms for each of data type can be seen in Figure 5.

### 4.2. Experiments on Different Datasets

First, the generalization of the proposed method is evaluated on three types of datasets in this section. The selected datasets are, respectively, the ship shafting vibration signals collected from the established ship shafting fault diagnosis experimental platform, as well as rolling bearing vibration signals from Case Western Reserve University (CWRU) and the rotor bearing laboratory of Xi’an Jiaotong University (XJTU-SY).

The specific information of the first dataset is detailed in Section 3.1. The second type of dataset sets a total of seven types of faults, with a sample length of 1024 and 350 samples for each type of data. The third type of dataset sets a total of five types of faults, with 300 samples for each type. The division ratio of the training set to the test set for all datasets is 7:3.

The learning rate is an important hyperparameter in the network training process. In order to obtain the optimal learning rate decay factor, this section first uses different learning rate decay factors to optimize the hyperparameter on the ship shaft system fault dataset. In this section, the decay factors selected for this experiment are 0.86, 0.88, 0.90, 0.92, 0.94 and 0.96. That is to say, the learning rate decays once according to the above ratio every three epochs. The specific principle is shown in Equation (9). In addition, in this experiment, the batch size is 32, the initial learning rate is 0.01, the reward discount factor γ is 0.001, the optimizer is Adam, and the total number of training epochs is 150.

**Figure 6:**
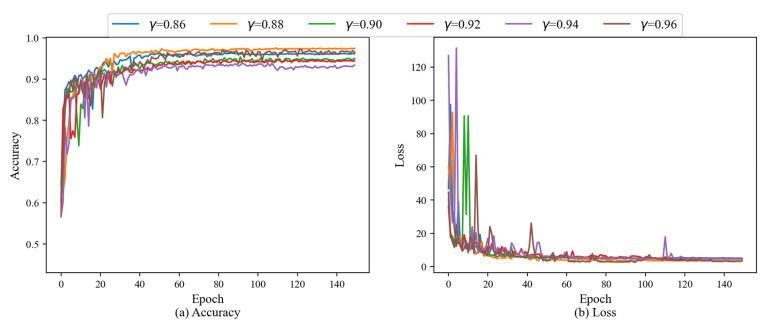
Accuracy and loss results under different learning rate decay factors.

The test accuracy and loss results are shown in Figure 6. Experimental results demonstrate that the proposed method can achieve high accuracy and low loss over all tested decay rates, which indicating that the agent effectively learned an optimal fault diagnosis strategy during training. Furthermore, the method exhibited the best performance with a decay rate of 0.88, at a test accuracy of 97.57%, and loss of 3.3274. Therefore, this paper selects this decay factor to adjust the learning rate in subsequent training.

**Figure 7:**
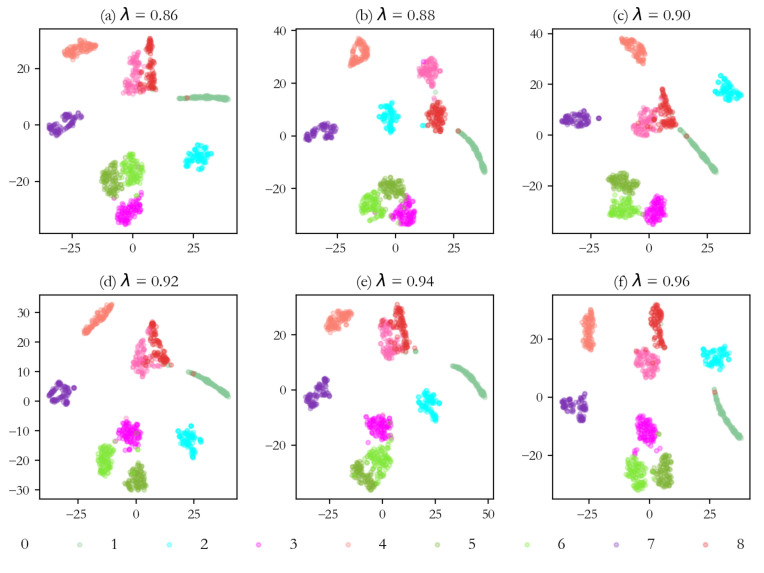
Visualization results of output features under different learning rate decay factors.

To further illustrate the experimental results, the t-distributed stochastic neighbor embedding (t-SNE) algorithm is used to visualize the output feature distribution of different fault modes under different learning rate decay factors. The visualization results of all the experiments are shown in Figure 7. It can be seen from Figure 7 that the proposed method has a good inter-class separation degree for each type of data under each decay factor, which demonstrates the superior generalization and robustness of the proposed method. Specifically, when the learning rate decay factors are 0.86, 0.88, and 0.96, the method has the highest inter-class separation on various data features. Meanwhile, the model performance is relatively similar in these cases, with classification accuracies of 96.41%, 97.57%, and 97.22% on the test set, respectively. When the learning rate decay factors are 0.90 and 0.92, the proposed method begins to produce slight confusion among different types of features, with accuracy of 95.37% and 94.79%, respectively. This indicates that the network has to some extent deviated from the optimal parameter in the gradient optimization process. When the decay factor is 0.94, the performance of the proposed method is the worst, with some confusion in multiple types of data features, and the corresponding accuracy and loss are 93.87% and 4.5653, respectively.

Finally, the generalization of the proposed method is evaluated on different datasets in this section. The specific experimental results are shown in Table 2. It can be seen from the results that the proposed method performs well on different datasets. Among them, it has the highest test accuracy on the second dataset and the smallest standard deviation on the first dataset, which indicates that the proposed method has better convergence stability on this dataset. It is also worth noting that the highest training accuracy of the proposed method on the second datasets is 1, which indicates the strong nonlinear fitting ability of the proposed method.

### 4.3. Ablation Experiment

**Figure 8:**
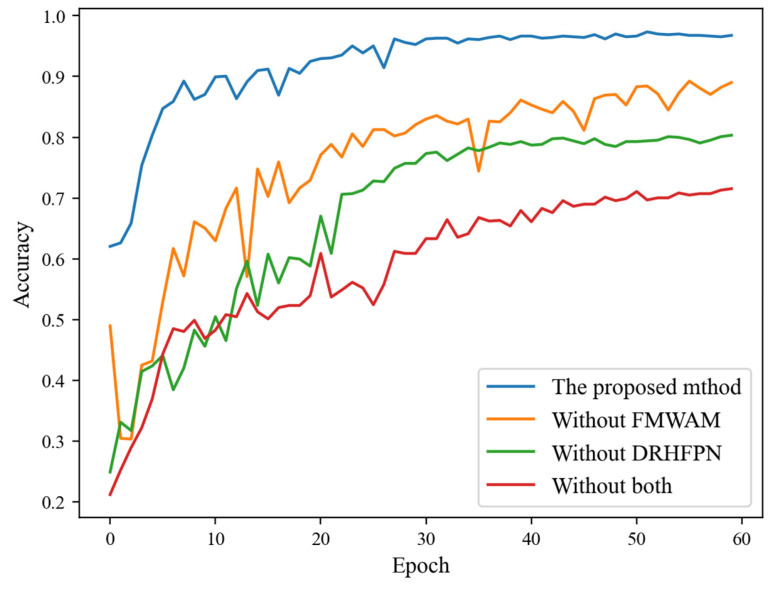
Experimental results of ablation experiments under different model structures.

In the previous section, the generalization of the proposed method was verified on different datasets. In this section, the effectiveness of the proposed modules is evaluated and its gain effect on fault diagnosis accuracy is quantified through ablation experiments. First, this paper quantifies the effects of FMWAM and DRHFPN in improving fault diagnosis accuracy. The experimental results are shown in Figure 8. It can be seen that the method proposed in this paper converges around the 30-th epoch, and its highest test accuracy is 97.57%. The first and second comparison structures removed FMWAM and DRHFPN, respectively, and their highest test accuracies were 89.24% and 80.32%, respectively, which decreased by 8.33% and 17.25% compared to the proposed method. The third comparison structure is to remove both FMWAM and DRHFPN. At this point, it became equivalent to an ordinary deep reinforcement learning model, and its performance degradation was the most severe, with the highest test accuracy being 71.53%.

**Figure 9:**
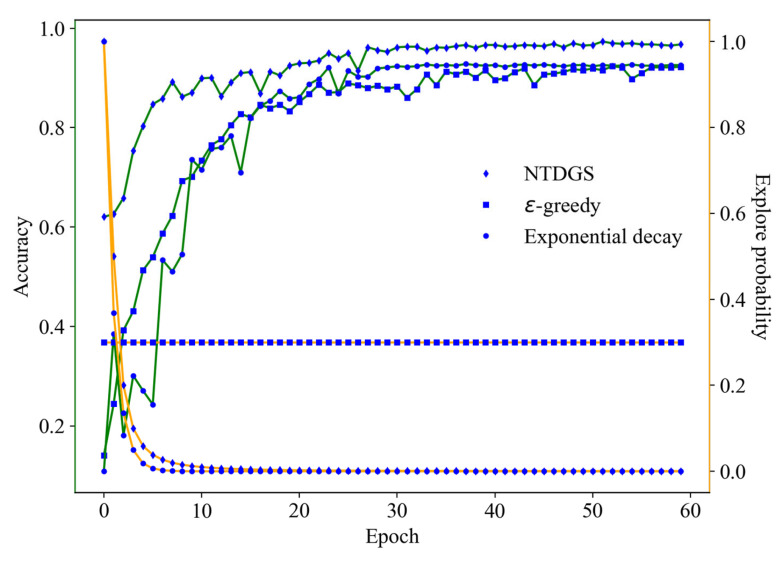
Experimental results of ablation experiments under different exploration strategies.

In addition, to further verify the effectiveness of NTDGS, NTDGS was compared with the epsilon-greedy and exponential decay methods. The experimental results are shown in Figure 9. As can be seen from the *Y*-axis on the left side of Figure 9, NTDGS has a faster convergence speed and higher convergence accuracy compared to the other two exploration strategies. Its accuracy reached 90.05% in the 12th epoch, while the other two exploration strategies were only 76.5% and 75.7%, respectively, at this point. Furthermore, the maximum test accuracies of the epsilon-greedy and exponential decay methods were 92.36% and 92.82%, respectively, which are 5.21% and 4.75% lower than NTDGS, respectively. Furthermore, as can be seen from the *Y*-axis on the right side of Figure 9, during the 5-th to 10-th epoch, NTDGS was more stable during the decay process compared to the exponential decay method. The exploration probability of epsilon-greedy remained unchanged throughout the network training process, which is equivalent to injecting additional noise in the later training process of the network. Finally, it is worth noting that independent tests have proven that NTDGS has an advantage over the exponential decay method in terms of computational efficiency. Specifically, the computational speed of NTDGS is approximately 3.55 times that of the exponential decay method.

### 4.4. Comparative Experiments of Different Neural Network Models

**Figure 10:**
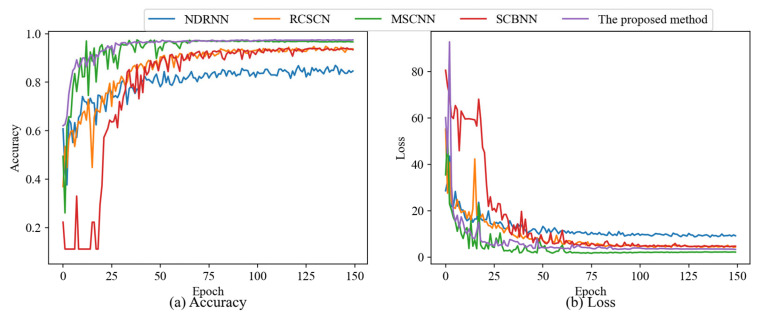
The training process of different network models. (**a**) The experimental results of accuracy. (**b**) The experimental results of the loss.

To further verify the effectiveness of the proposed method, four classical neural network models were selected for comparison in this section. Moreover, in order to enhance the challenge of comparative experiments, traditional baseline methods such as residual network (ResNet) and convolutional neural network (CNN) were optimized by adding advanced network modules like multi-scale convolution, dense residual block, channel shuffle, and normalized feature fusion mechanisms to them. The comparison models included the residual channel shuffle convolutional network (RCSCN), normalization dense residual neural network (NDRNN), sequence convolutional block neural network (SCBNN), and multi-scale convolutional neural network (MSCNN). Each model was trained in the same number of epochs. The accuracy and loss results for each method on test set are presented in Figure 10.

As shown in Figure 10, the proposed method outperformed all of the comparison models, achieving the highest test accuracy of 97.57% with a corresponding loss of 3.497374 at convergence. MSCNN demonstrated slightly lower overall performance, reaching a maximum test accuracy of 97.45%, and exhibited a slower convergence rate compared to the proposed method. The SCBNN and RCSCN also showed marginally inferior test accuracy and training stability, with test accuracies of 94.10% and 94.80%, respectively, indicating a somewhat limited capability in fault feature identification and classification. NDRNN displayed the poorest overall performance in this experiment, with a maximum test accuracy of only 86.92%, suggesting that its feature recognition ability requires further improvement.

**Figure 11:**
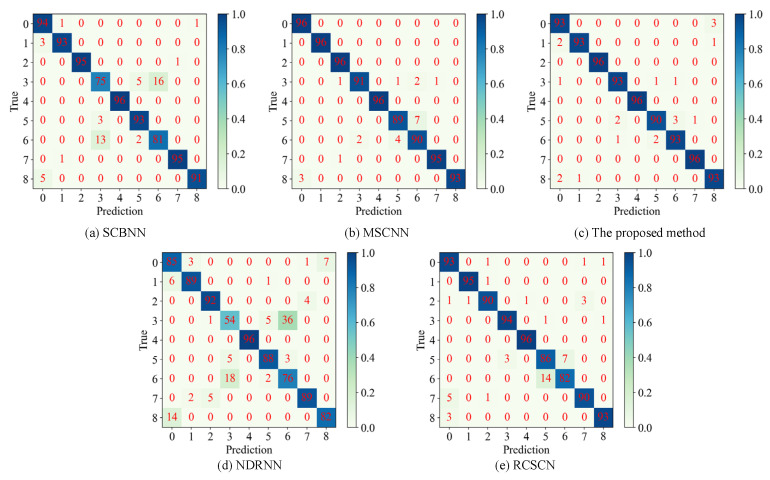
The specific classification of each type of data on test set by different methods in a certain experiment.

To further illustrate the experimental results, confusion matrices for each method on test data were plotted. It can be seen from Figure 11 that MSCNN exhibited slightly inferior performance compared to the proposed method, with some sixth-class samples being misclassified as seventh-class samples, resulting in a recognition accuracy of only 92.7% for the sixth-class test data. This misclassification is attributed to the high similarity in feature distribution between the sixth and seventh data types, indicating the method’s limited ability to distinguish easily confusable samples. SCBNN performs poorly on both the fourth and seventh data types, with accuracy rates of 78.1% and 84.4%, respectively. Similarly, RCSCN shows subpar performance on the sixth and seventh data types, achieving accuracy rates of 89.6% and 85.4%, respectively, suggesting an inability to effectively learn the hidden high-level abstract fault features of these data types. NDRNN demonstrates the weakest overall performance in this comparative experiment, further supporting its limited capability in abstract feature extraction, as evidenced by the results shown in Figure 10. In contrast, the proposed method achieved robust performance across all data samples with a highest test accuracy of 97.57%, thereby validating its effectiveness.

### 4.5. Anti-Noise Experiment at Different Signal Noise Ratios

In this section, the anti-noise performance of the proposed method was evaluated on datasets with varying signal noise ratios (SNRs) to comprehensively demonstrate the effectiveness of the proposed method in strong-noise environments. Specifically, the noise resistance of each comparison method was tested using experimental data with SNRs of 2, 4, 6, 8, 10, and 12 dB. The specific formula used to calculate the SNR of the experimental data is as follows:

(11) $$SNR=10\log_{10}\frac{P_{s}}{P_{n}}$$

where $P_{s}$ and $P_{n}$ indicate the effective power of original signal and noise data.

**Figure 12:**
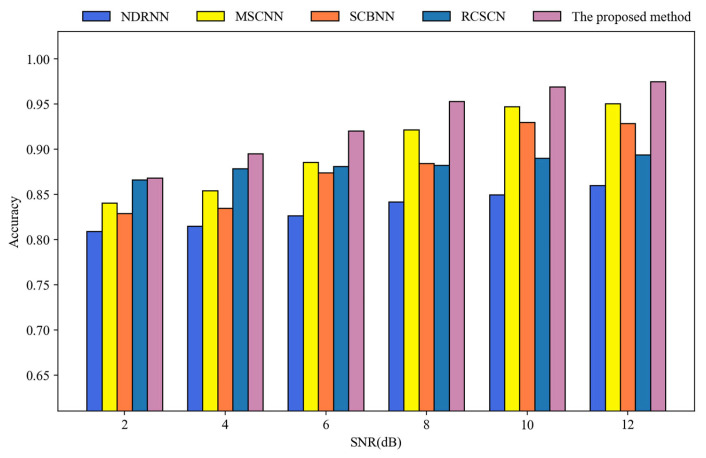
Experimental results of all the comparison methods under different strong noise conditions.

The experimental results of each method across different SNRs are presented in Figure 12. The proposed method demonstrates superior performance in all the strong noise scenarios, maintaining an accuracy of up to 86.81% even when SNR is as low as 2 dB. MSCNN performs well on datasets with lower noise interference, achieving accuracy levels slightly below those of the proposed method. However, as noise interference increases, its feature recognition ability diminishes rapidly, indicating limited robustness in handling strong-noise data. Similarly, SCBNN shows a gradual decline in test accuracy as noise interference intensifies, suggesting an inability to effectively resist the impact of strong noise on network recognition. RCSCN exhibits slightly better overall anti-noise performance than NDRNN but still falls short compared to other methods, indicating room for improvement in noise processing capabilities. NDRNN consistently performs the worst in various strong noise scenarios, thereby demonstrating a lack of capacity for processing the signals with strong background noise.

Finally, the experimental results of each method were compared comprehensively, as shown in Table 3. As shown in Table 3, the proposed method consistently outperforms the others across all strong noise environments in terms of fault diagnosis accuracy, demonstrating its superior noise resistance. However, the computational speed of the proposed method is slightly inferior to that of RCSCN and NDRNN, and further improvement is needed in future research. Whereas MSCNN and SCBNN perform well on the data with high SNR, their performance significantly deteriorates in strong-noise environments, indicating an inability to effectively process noisy data. Additionally, these two methods have the highest number of parameters compared to other methods and are not as fast in terms of computational speed as other methods. RCSCN and NDRNN have a similar number of network parameters and computational speeds. However, RCSCN exhibits slightly better overall performance than NDRNN in the experiment. NDRNN has the worst performance in noise resistance, and its performance on the strong-noise data is not as good as other methods, indicating that this method has little fault diagnosis ability on strong-noise data.

## 5. Conclusions

In response to the issues such as strong background noise and limited computing resources in practical industrial scenarios, a novel noise-resistant and lightweight fault diagnosis framework with nonlinear timestep degenerative greedy strategy (NTDGS) and dual residual horizontal feature pyramid network (DRHFPN) is proposed in this paper for fault diagnosis in strong noise scenarios. In the proposed method, NTDGS is constructed to balance the agent’s exploration and utilization of knowledge at different training stages, optimizing the overall training objective of the network and enhancing the stability in the process of training the network. In addition, DRHFPN is designed to filter out the strong background noise hidden in the original vibration signals by extracting and fusing different fine-grained features, which enhances the network’s robustness and noise resistance. Finally, FMWAM is developed to enhance the network’s ability to extract weak features in strong noise scenarios. The generalization and noise resistance of the proposed method are verified in different datasets and different strong noise environments. Experiments show that compared with other advanced fault diagnosis methods, the proposed method has better noise resistance, higher fault diagnosis accuracy, and fewer parameters.

In future research, we will further optimize the structure of the proposed method to improve its computational speed and expand its application scope to more challenging and practical scenarios, such as small sample fault diagnosis in strong noise scenarios. In addition, we will also consider building a universal multi-task framework by combining large language models to simultaneously handle tasks such as fault diagnosis and life prediction.

## Figures and Tables

**Table 1:** Experimental dataset.

Label	Machine Condition	Training Sample Size	Testing Sample Size
0	Normal Condition	224	96
1	Unbalance Fault	224	96
2	Looseness Fault	224	96
3	0.1 mm Unilateral Misalignment Fault	224	96
4	0.1 mm Bilateral Misalignment Fault	224	96
5	0.3 mm Unilateral Misalignment Fault	224	96
6	0.3 mm Bilateral Misalignment Fault	224	96
7	0.5 mm Unilateral Misalignment Fault	224	96
8	0.5 mm Bilateral Misalignment Fault	224	96

**Table 2:** Experimental results on different datasets.

Dataset	Train Accuracy	Train Loss	Test Accuracy	Test Loss
Dataset I	1.00 ± 0.0011	0.0557 ± 0.2868	0.9757 ± 0.0010	3.3274 ± 0.0359
Dataset II	1.00 ± 0.0027	0.0667 ± 15.3333	0.9960 ± 0.0009	0.6271 ± 2.8911
Dataset III	1.00 ± 0.0037	0.3552 ± 76.1892	0.9800 ± 0.0026	0.8447 ± 16.5105

**Table 3:** Numerical experimental results of all the comparison methods under different strong noise conditions.

Methods	Parameters	SNRs						Runtime
		2 dB	4 dB	6 dB	8 dB	10 dB	12 dB	
NDRNN	897,641	80.90 ± 0.0217	81.48 ± 0.0170	82.64 ± 0.0147	84.14 ± 0.0257	84.95 ± 0.0157	85.60 ± 0.0088	0.161 s
MSCNN	1,005,833	84.03 ± 0.0307	85.42 ± 0.0147	88.54 ± 0.0252	92.13 ± 0.0073	94.68 ± 0.0089	95.02 ± 0.0399	0.339 s
SCBNN	1,442,953	82.87 ± 0.0219	83.45 ± 0.0439	87.38 ± 0.0240	88.43 ± 0.0481	92.94 ± 0.0261	92.82 ± 0.0363	0.267 s
RCSCN	650,649	86.57 ± 0.0093	87.85 ± 0.0194	88.08 ± 0.0113	88.19 ± 0.0173	89.00 ± 0.0183	89.35 ± 0.0089	0.119 s
The proposed method	485,900	86.81 ± 0.0040	89.47 ± 0.0056	92.01 ± 0.0028	95.25 ± 0.0010	96.88 ± 0.0007	97.45 ± 0.0186	0.263 s

## Data Availability

The raw data supporting the conclusions of this article will be made available by the authors on request.
